# Confinement Effect of Plasmon for the Fabrication of Interconnected AuNPs through the Reduction of Diazonium Salts

**DOI:** 10.3390/nano11081957

**Published:** 2021-07-29

**Authors:** Luong-Lam Nguyen, Quang-Hai Le, Van-Nhat Pham, Mathieu Bastide, Sarra Gam-Derouich, Van-Quynh Nguyen, Jean-Christophe Lacroix

**Affiliations:** 1 Department of Advanced Materials Science and Nanotechnology, University of Science and Technology of Hanoi (USTH), Vietnam Academy Science and Technology, 18 Hoang Quoc Viet, Cau Giay, Hanoi 100000, Vietnam; nguyen-luong.lam@usth.edu.vn (L.-L.N.); quanghaile8396@gmail.com (Q.-H.L.); pham-van.nhat@usth.edu.vn (V.-N.P.); 2 Chemistry Department, Université de Paris, ITODYS, UMR 7086 CNRS, 15 Rue Jean-Antoine de Baïf, CEDEX 13, 75205 Paris, France; mathieu.bastide92@gmail.com (M.B.); sarra.derouich@univ-paris-diderot.fr (S.G.-D.)

**Keywords:** interconnected gold nanoparticles, plasmonic electrochemistry, diazonium reduction, nanofabrication, hot electrons

## Abstract

This paper describes a rapid bottom-up approach to selectively functionalize gold nanoparticles (AuNPs) on an indium tin oxide (ITO) substrate using the plasmon confinement effect. The plasmonic substrates based on a AuNP-free surfactant were fabricated by electrochemical deposition. Using this bottom-up technique, many sub-30 nm spatial gaps between the deposited AuNPs were randomly generated on the ITO substrate, which is difficult to obtain with a top-down approach (i.e., E-beam lithography) due to its fabrication limits. The 4-Aminodiphenyl (ADP) molecules were grafted directly onto the AuNPs through a plasmon-induced reduction of the 4-Aminodiphenyl diazonium salts (ADPD). The ADP organic layer preferentially grew in the narrow gaps between the many adjacent AuNPs to create interconnected AuNPs. This novel strategy opens up an efficient technique for the localized surface modification at the nanoscale over a macroscopic area, which is anticipated to be an advanced nanofabrication technique.

## 1. Introduction

Advances in miniaturization technologies have attracted enormous interest due to their effectiveness in the fabrication of smaller, faster, and cost-effective devices. Various nanosized multifunctional surfaces have been manufactured for sensing systems [1,2,3,4], switching electronic devices [5,6], light-emitting systems [7] and energy storage devices [8]. Nanostructured surfaces are fabricated by using either top-down or bottom-up approaches. The conventional top-down methods, such as photolithography [9,10] and e-beam lithography [11] require several steps to generate a structured surface. The fabrication processes based on top-down methods remain time-consuming, expensive, require complicated facilities and show limited resolution [12]. They cannot be the only response to the growing demands for product miniaturization.

On the other hand, bottom-up approaches, such as 3D direct laser printing [13], nanosphere lithography combined with electrochemical deposition [14,15,16,17] generate nanostructures [18,19,20]. These fabrication approaches allow the construction of the desired nanostructures in a range from 2 to 10 nm, which is not only crucial for the continued miniaturization of devices, but is also promising for a variety of novel applications in biosensing, catalysis [21], and optics [22,23,24,25,26].

Nanosized metallic particles (i.e., silver, gold, copper) have the efficient capability of trapping photons based on localized surface plasmon resonance (LSPR) [27,28]. Light−matter interaction on the plasmonic surfaces is significantly optimized by confining the light energy in a tiny region of the enhanced electromagnetic field [29,30]. As a consequence of plasmon excitation, hot charged carriers including hot electrons and holes are generated accordingly [31,32,33]. Due to the combination of the local electromagnetic field enhancement, the increased temperature and the generated carriers with sufficient energy caused by plasmon excitation, some chemical and redox reactions can be triggered. The evidence of the plasmon-induced chemical reaction has been widely reported, both in experiment and in theory [33,34,35,36,37,38]. However, the mechanism of such reactions has not been fully distinguished. There are also still various debates concerning the distinction between the plasmon-induced charge transfer phenomena and co-catalysis effect, plasmonic heating effect and nanoantenna effect [39]. The reaction can be photo-induced by either the enhanced electric field near the plasmonic NPs, or hot carriers (i.e., electrons and holes) generated from plasmon decaying transfer directly to the appropriate electronic states of nearby molecules, or by the increase of local heating due to light irradiation, or any combination of them. Despite the reaction mechanism still being unclear, the metallic NP-based plasmonic substrate has proved its potential for triggering specific redox reactions based on the LSPR. Because the enhanced electromagnetic field, hot carrier generation and thermal effects are spatially localized, irradiation of plasmonic substrates opens the possibility to regio selectively functionalize the surface.

Recently, many studies on the topic of plasmon-driven chemical reaction have been reviewed [40,41,42], including the reduction of nitro-aromatic compounds on supported AuNPs illuminated by a low-intensity UV light [43]. However, the plasmon-driven reduction reaction has shown a lower efficiency than those with additional photocatalysts inside. Hence, improving the catalytic efficiency of plasmon-driven chemical reactions and their applications on a large scale need further studies in the prospective future. We have strong confidence that the unique characteristics of plasmons will draw more and more attention and that adds a novel route for advanced techniques in nanofabrication.

**Figure 1:**
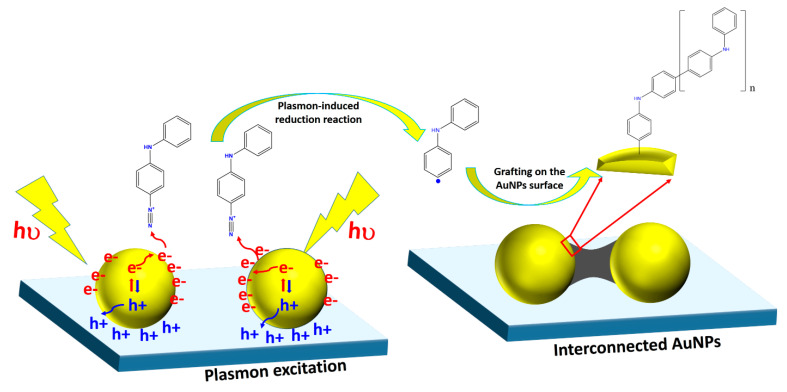
Scheme of the plasmon-induced grafting process of ADP oligomers on AuNPs.

This paper demonstrates experimentally the plasmon-induced reduction of 4-Aminodiphenyl diazonium salts and the deposition of oligo (4amino-diphenyl) (ADP) at the hotspot region of AuNPs deposited on the ITO surface (Figure 1). The key element for driving this reaction is supposed to be the hot electrons generated by the plasmonic substrate and transferred to the diazonium salts absorbed on the metal surface [34]. We show that the ADP organic layer preferentially grows in narrow gaps between some adjacent AuNPs to create interconnected AuNPs. The confinement effect of the LSPR phenomena at the AuNP surface allows the fabrication of structures that are rarely achieved by either self-assembly or self-organization and opens a rapid bottom-up approach for functionalizing selectively AuNPs on transparent substrates.

## 2. Materials and Methods

All chemicals such as 4-Amino-diphenylamine molecules (ADPA), Tetrabutylammonium tetrafluoroborate (TBABF_4_), perchloric acid (HClO_4_), sodium nitrite (NaNO_2_), potassium gold(III) chloride (KAuCl_4_), sodium carbonate (Na_2_CO_3_), hydrogen peroxide 30% (H_2_O_2_) and ammonium hydroxide solution (NH_4_OH) were purchased from Sigma-Aldrich, Singapore. They were analytical grade and were used directly without any further purification. Indium tin oxides (ITO) substrates were supplied by LUMTEC, Taipei City, Taiwan.

### 2.1. Generation of the Plasmonic Substrate by Electrochemical Deposition of Spherical AuNPs

The ITO substrates of 2 × 2 cm^2^ were firstly sonicated in a cleaning solution that is a mixture of H_2_O/NH_4_OH/H_2_O_2_ (5:1:1 by volume) for 20 min, then were rinsed thoroughly with distilled water. Finally, they were stored in ultrapure Milli-Q water until use.

The AuNPs were electrochemically deposited on a working electrode of ITO by the chronoamperometry (CA) technique with a three-electrode cell autolab potentiostat instrument (i.e., Metrohm-Australia ). The Ag/AgCl reference electrode and the Pt counter electrode were utilized. In these experiments, a solution of 0.012 mM [AuCl_4_] and 0.015 M Na_2_CO_3_ was prepared and held under argon gas during the deposition procedure.

### 2.2. Plasmon-Induced Grafting of ADP on AuNP Surface

A solution of 20 mL of ADPA 3 mM was prepared by dissolving the ADPA in Milli Q water with HClO_4_ 0.1 M; The mixture solution was then ultrasonicated for 10 min at room temperature for dissolution and dispersion. Half of the ADPA+HClO_4_ solution (~10 mL) was extracted and degassed with Argon for 10 min before generating diazonium salts. Diazotization reaction was carried out by injecting 60 µL NaNO_2_ 1 M, which corresponded to 2eQ of NaNO_2_. The solution color changed from light blue to red-brown, after 10 min of the diazotization reaction. In the following steps, 3 mL of diazonium salt solution was placed in the handmade electrochemical cell, with the working electrode of AuNPs/ITO set as the bottom window (Figure S1). The cell was then irradiated for 15 min by a lamp integrated inside the microscope for performing a photochemical reaction. Finally, the AuNPs/ITO electrode was thoroughly rinsed with Milli Q water and dried at room temperature before characterizing by Scanning electron microscopy SEM (Zeiss SUPRA 40 from Carl Zeiss AG, Germany) and UV−VIS spectrometer (USB4000-VIS-NIR- Ocean Optics, Inc., Germany).

## 3. Results

To generate plasmonic substrates for studying the reduction of diazonium salts triggered by plasmon, spherical AuNPs were electrochemically deposited on transparent ITO electrodes using a well-established procedure [44]. This one-step template-free electrochemical process allows isolated AuNPs to be deposited on a large area of the electrode (i.e., ~several cm^2^) in less than 100 s. Whereas a small area (i.e., 10^−4^ cm^2^) of AuNP array generated by the E-beam lithography technique arrays requires several fabrication steps, along with being expensive and time-consuming [45].

Interestingly, with the electrochemical approach, many sub-30 nm spatial gaps between the deposited AuNPs can be randomly generated on the substrate, which is not easy to obtain via a top-down approach like E-beam lithography, due to its fabrication limits. These tiny spatial gaps are well recognized as hotspots which play a significant role [46] in investigating local field enhancement caused by the LSPR phenomena. Hence, all plasmonic substrates in this paper will be prepared by electrochemical deposition. 

After the deposition of AuNPs on the ITO electrode, the shape and size of the deposited AuNPs were characterized by scanning electron microscopy (SEM) and UV−Vis absorption.

**Figure 2:**
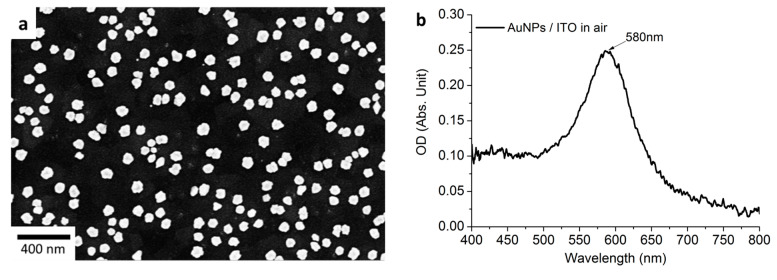
(**a**) The SEM image of deposited AuNPs and (**b**) corresponding extinction spectra.

The SEM image (Figure 2a) shows a random distribution of AuNP with the size of 50–55 nm in diameter on the electrode surface. Many tiny spatial gaps (i.e., between 10 and 30 nm) between two adjacent particles were observed. Despite the lack of organization, this as-prepared AuNP substrate had a strong absorption peak located at 580 nm in air that was attributed to the localized surface plasmon resonance (LSPR) of the AuNPs.

Next, the as-prepared plasmonic substrates were plugged into a solution of 4-Aminodiphenyl diazonium (ADPD), which was prepared by adding 2eQ of NaNO_2_ into a 10 mL aqueous solution of 4-Aminodiphenylamine (ADPA) 3 mM and HClO_4_ 0.1 M. Photoelectrochemical reactions (called plasmonic grafting) were performed subsequently by visible light irradiation for 15 min. 

Two other controlled experiments (i.e., electrochemical grafting and spontaneous grafting in the dark) were carried out to graft ADP molecules onto the electrode surface, to compare the results to those obtained by plasmonic grafting in 15 min. 

**Figure 3:**
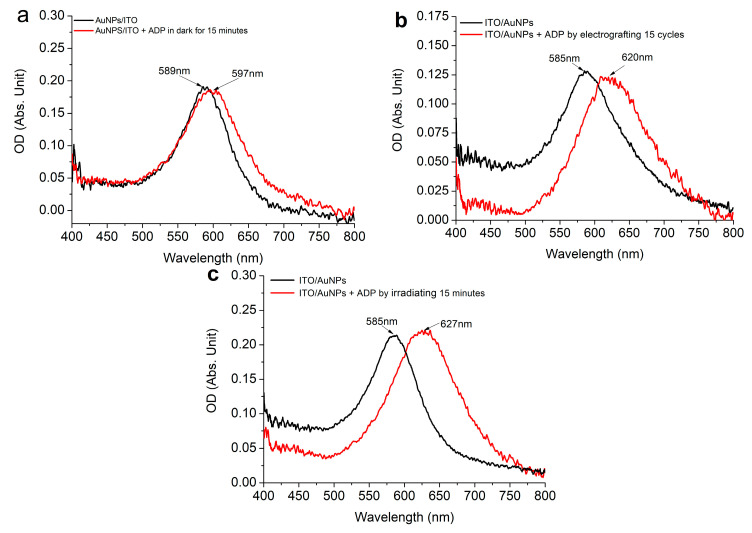
Extinction spectra of deposited AuNPs/ITO before and after (**a**) 15 min of spontaneous grafting in the dark; (**b**) 15 cycles of grafting by electrochemical grafting; (**c**) 15 min of irradiation by visible light.

Figure 3 shows the extinction spectra of deposited AuNPs/ITO before and after 15 min of spontaneous grafting in the dark (Figure 3a), 15 cycles of grafting by electrochemical grafting (Figure 3b) and 15 min of irradiation by visible light (Figure 3c). In all figures, a similar red shift upon grafting ADP molecules on AuNPs/ITO substrates was observed. These red shifts indicate that the dielectric constant of the AuNPs surrounding media has changed and are consistent with previous findings for the grafting of the bis-thienyl benzene (BTB) molecules on the plasmonic substrate [34]. A red-shift of 8 nm, 35 nm and 42 nm was observed for the three different experiments to graft ADP by different approaches, such as spontaneous grafting (Figure 3a), electro grafting (Figure 3b) and plasmonic grafting (Figure 3c), respectively. These results demonstrate the existence of an ADP layer covering the AuNPs in all three distinct cases. Moreover, the thickness of the ADP grafted layer correlates with the magnitudes of the shift of the LSPR peak. A red-shift of 8 nm (Figure 3a) may correspond to an ADP layer with an estimated thickness of only 2−3 nm [47] that spontaneously grafted around AuNPs for 15 min in the dark. It is much thinner than that obtained by either electrochemistry or the irradiation method. A red-shift of 35 nm (Figure 3b) and 42 nm (Figure 3c) indicates an estimated thickness of the ADP layer is close to 20 and 25 nm, respectively.

**Figure 4:**
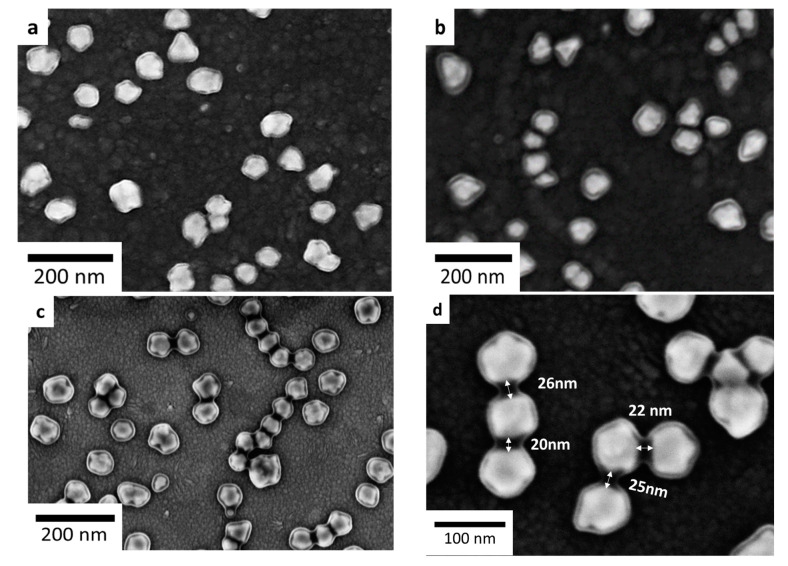
SEM images of AuNP-based plasmonic substrate after (**a**) 15 min of spontaneous grafting in the dark; (**b**) 15 cycles of electrochemical grafting; (**c**,**d**) 15 min of irradiation by visible light.

The SEM images (Figure 4) show the physical appearance of isolated AuNPs with a random distribution on the surface. Each individual AuNP has a different contrast between the inside and the outside. The brighter area inside is attributed to AuNP while the darker region outside could be the ADP layer because AuNPs are obviously more conductive than the organic layer of ADP.

**Figure 5:**
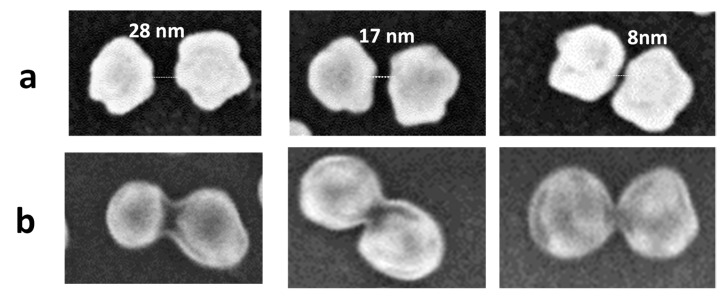
(**a**) AuNP dimers separated by gaps ranging from 8 to 30 nm. (**b**) AuNP dimer connected by an oligo (ADP) layer after irradiation of the plasmonic electrode in 4-Aminodiphenyl diazonium solution for 15 min.

Figure 4a shows clearly a very thin layer of ADP coated around AuNP, while Figure 4b,c show a thicker organic layer covering the AuNP. These results are in good agreement with those discussed in Figure 3. Interestingly, each separated AuNP in Figure 4a,b appeared to be surrounded by a homogeneous layer. It indicates that the ADP layers grafted by spontaneous deposition (Figure 4a) and electrochemical deposition (Figure 4b) are mainly the result of isotropic growth. Besides, Figure 4c shows more localized and anisotropic growth of the ADP layer around AuNPs under the irradiation condition. The ADP layer was deposited on the AuNPs with no apparent deposition on the underlying ITO substrate. It was also preferentially deposited in the narrow gaps created by some nearby AuNPs, while isotropic growth still occurred locally around isolated AuNPs. As a consequence, two neighboring AuNPs, separated by gaps ranging from 10 to 30 nm, appeared to be interconnected as depicted in Figure 4c,d. Figure 5 and Figure S2 and several adjacent AuNPs may be linked by a string of ADP layers. 

**Figure 6:**
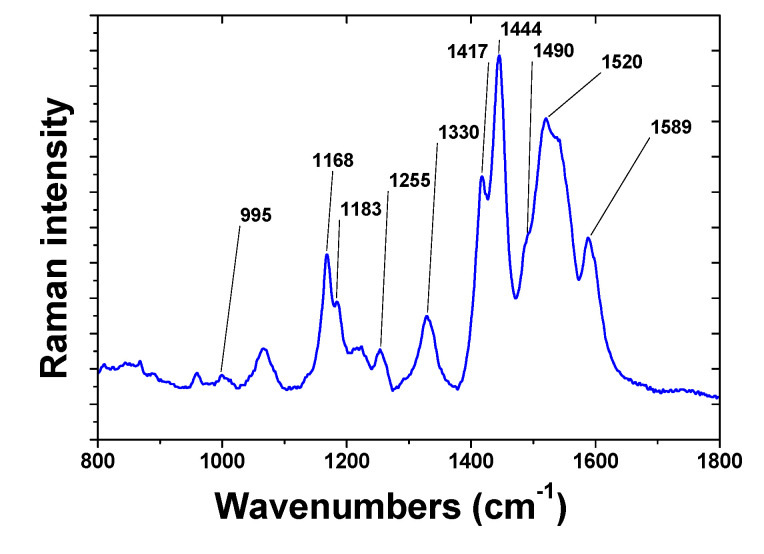
Raman spectrum of the organic film deposited on a AuNPs/ITO electrode by plasmon induced grafting from a ADPA diazonium solution.

The organic layer deposited on the plasmonic substrate was also analyzed by surface enhanced Raman spectroscopy (SERS) (Figure 6) using laser excitation at 633 nm. The SERS spectrum showed several Raman shifts which had already been observed in the Raman spectra of polyaniline (PANI) deposited by electrochemical oxidation of aniline [48,49]. The C=C and C=N shifts at 1590 and 1520 cm^−1^ were very strong and indicated the presence of phenyl on the surface. Raman shifts between 1400 and 1500 cm^−1^ were also clearly visible and were the already observed SERS spectra of PANI deposited on rough Ag or Au electrodes [50]. These bands could also be attributed to azo groups that could remain in the films [51]. Intense bands at 1255 and 1170 cm^−1^ also observed are probably due to in-plane C–H bending of aromatic units known to be strong in Raman spectroscopy. Such spectra indicate that (i) the AuNPs are covered by an π-conjugated organic material; (ii) this π-conjugated organic material has a structure similar to that of oligoaniline.

Overall, this work demonstrates that under visible irradiation, ADP can be directly grafted onto AuNPs or in between AuNP without using any photocatalyst in the system, thanks to plasmon-induced electrochemistry.

The most probable mechanism remains a plasmon-induced reduction of the diazonium salt even though a thermal effect cannot be ruled out. Plasmon decaying through Landau damping, occurring within 1–100 fs of light irradiation [31] can excite electrons to new energy levels that are higher than the initial fermi level of the gold. These hot electrons with sufficient energy may be transferred, concertedly or subsequently to light absorption, to the unoccupied state of adsorbed molecules (i.e., LUMO—lowest unoccupied molecular orbital). This leads to the occurrence of a reduction reaction of molecules on the gold surface.

In the present case, when the AuNPs with a work function (WF) of 5.1 eV [52] came into contact with the ITO substrate (WF = 4.7 eV) [53], the free-electrons on the ITO substrate transferred to the AuNPs, due to the WF difference. The electron transfer terminates when the Fermi level of AuNPs and ITO are aligned. In these circumstances, a Helmholtz double layer has formed at the AuNPs/ITO interface, with the AuNPs surface negatively charged, while the ITO surface is positively charged near its surface due to the electrostatic interaction. As a result, the free electron population of AuNPs increases. Under irradiation for plasmon excitations, generated hot electrons are probably transferred directly to the LUMO of ADP molecules inducing the reduction of ADPD on AuNPs. Consequently, the experimental results (Figure 4c) clearly showed anisotropic growth of ADP in between a series of adjacent AuNPs resulting in the formation of dimers or chains of interconnected AuNPs by an organic layer.

This section is divided by subheadings. It should provide a concise and precise description of the experimental results, their interpretation, as well as the experimental conclusions that can be drawn.

## 4. Conclusions

This paper uses a rapid bottom-up approach to generate a cheap and high performance plasmonic substrate on a large scale, using the one-step template-free electrochemical process. These plasmonic substrates have been used to investigate the plasmon-driven chemical reaction of 4-Aminodiphenyl diazonium salts. We show that localized plasmons can be used as a stable and efficient “photocatalyst” for the reduction of ADPD diazonium salts in the vicinity of spherical AuNPs and in the gaps of various AuNP dimers, where the two AuNPs are separated by gaps between 10 and 30 nm. Thanks to the confinement effect of the LSPR phenomena at hotspots, the microfabrication of AuNP dimers bridged by, or of AuNP strings interconnected by, an ADP layer with sizes below 30 nm has been demonstrated experimentally. This fabrication approach will have a significant impact in manufacturing molecular circuitry and nanophotonic devices in the future. 

## Data Availability

Data is contained within the article or Supplementary Materials.

## Supplementary Materials

The following are available online at https://www.mdpi.com/article/10.3390/nano11081957/s1, Figure S1: Setup used for plasmon-induced chemistry; Figure S2: Other AuNP dimers connected by a layer of oligo(ADP) generated by irradiation of a plasmonic electrode in 4-Aminodiphenyl diazonium solution for 15 min.
