# Supplementary material for: Confinement Effect of Plasmon for the Fabrication of Interconnected AuNPs through the Reduction of Diazonium Salts
Source: Nanomaterials (Basel). 2021 Jul 29;11(8):1957. doi: 10.3390/nano11081957 (PMC8397949; doi:10.3390/nano11081957)
Supplement: Supplementary file 1 [file nanomaterials-11-01957-s001.zip › nanomaterials-1289343-supplementary.pdf]

nanomaterials

Supporting information

Confinement Effect of Plasmon for the Fabrication of Interconnected AuNPs Through the Reduction of Diazonium Salts

Luong-Lam Nguyen [1], Quang-Hai Le [1], Van-Nhat Pham [1], Mathieu Bastide [2], Sarra Gam-Derouich [2], Van-Quynh Nguyen [1,*] and Jean-Christophe Lacroix [1,2,*]

[1] Department of Advanced Materials Science and Nanotechnology, University of Science and Technology of Hanoi (USTH), Vietnam Academy Science and Technology, 18 Hoang Quoc Viet, Cau Giay, Hanoi, Vietnam
[2] Chemistry Department, Université de Paris, ITODYS, UMR 7086 CNRS, 15 rue Jean-Antoine de Baïf, 75205 Paris Cedex 13, France
* Correspondence: nguyen-van.quynh@usth.edu.vn (V.-Q.N.); lacroix@u-paris.fr (J.-C.L.)

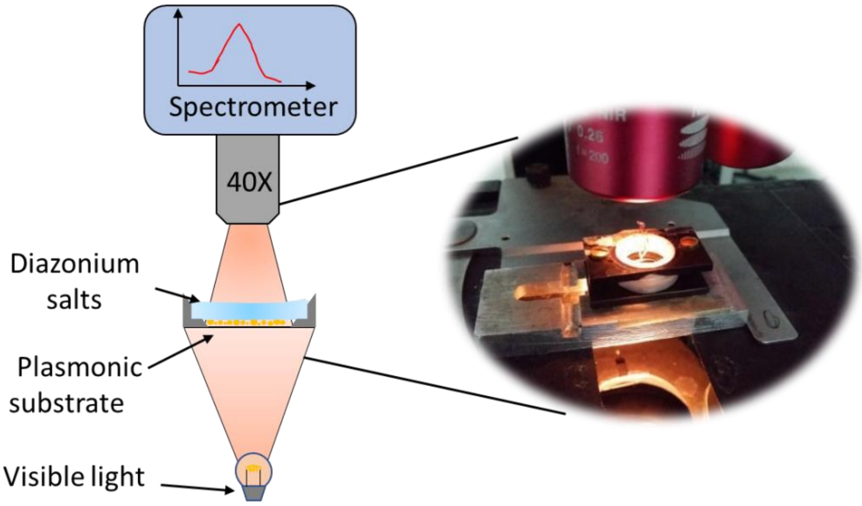

Figure S1. Setup used for plasmon-induced chemistry.

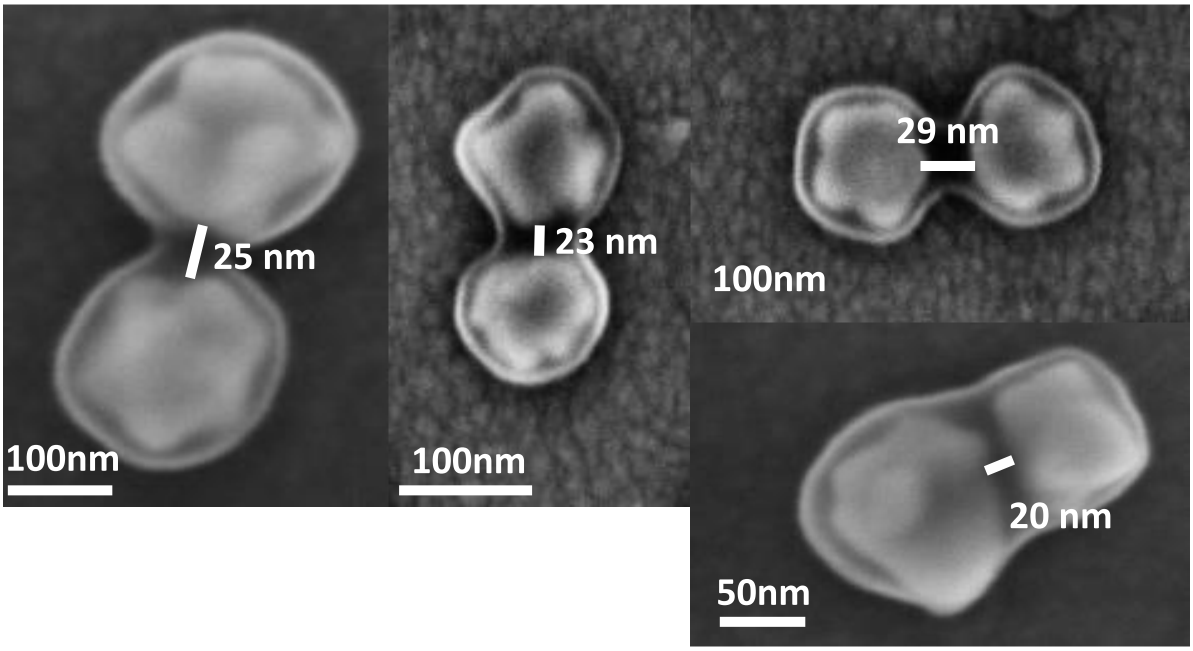

Figure S2. Other AuNP dimers connected by a layer of oligo(ADP) generated by irradiation of a plasmonic electrode in 4-Aminodiphenyl diazonium solution for 15 minutes.
